# Hahnemann’s Tomb

**Published:** 1898-04

**Authors:** Leon Brasol

**Affiliations:** St. Petersburg, Nikolaievskaia, 8


### HAHNEMANN'S TOMB.

St. Petersburg, Nikolaievskaia, 8,
28 February, 1898.

Bushrod W. James, M. D.

My Dear Doctor: I now have the opinion of all our members, and send you the appeal of the committee in the final form and ready for publication. The majority of votes in the committee—Drs. Cartier, Villers, and I—to which you also have given your adhesion, consider it undesirable to exclude Americans from the subscriptions, seeing that on the committee there is a representative of the United States, and that the subscription in that case would no longer be international.

You have probably already received from Dr. Cartier the letters of invitation to the homœopathic societies, and now it remains to proceed to the collection of pecuniary means without loss of time, of which there is now little left.

I hope that you will apply all your influence for the successful collection of contributions.

Our Society of Homœopathic Physicians, of which I am the President, has assigned 2,000 francs. With best wishes, I remain,

Yours sincerely,

Dr. Leon Brasol.

